# Study on the effect of porosity of hollow fiber membrane on humidification performance

**DOI:** 10.1038/s41598-022-07869-y

**Published:** 2022-03-09

**Authors:** Runping Niu, Xiaoting Jia, Lizhi Geng

**Affiliations:** School of Environment and Energy Engineering, Beijing University of Civil Engineering and Architecture, Beijing, 100044 China

**Keywords:** Materials for devices, Structural materials

## Abstract

Hollow fiber membranes are used in industrial processes widely. Porosity is one of the important parameters affecting the humidification performance of hollow fiber membrane components. The aim of this study was to analyze the effect of porosity of hollow fiber membrane on humidification performance. In order to perform this analysis, a model based on the finite element method was used to simulate numerically the heat and mass transfer under 6 porosity conditions. Five working conditions with different air flow was considered in order to get more data. The results show that when the porosity increases from 0.35 to 0.8, the humidification performance is greatly improved. However, when it increases from 0.8 to 0.9, the humidification performance is almost unchanged. Considering the humidification performance and support strength of hollow fiber membrane, it is suggested to control the porosity of hollow fiber membrane between 0.65 and 0.8.

## Introduction

Indoor air humidity is not only directly related to human comfort but also closely related to human health. Air with too little or too much humidity can lead to decreased comfort, and even cause mouth or eye dryness, respiratory tract infection and other diseases^1^. In an industrial production environment, the scientific regulation of humidity control in a reasonable range of humidity also has a vital position. If humidity is not be regulated, it will seriously affect the product quality and cause unnecessary economic losses, such as in electronic components, food processing, wood furniture, agricultural production and other industries.

There are cooling dehumidification, liquid absorption dehumidification, solid adsorption dehumidification, membrane dehumidification, membrane liquid dehumidification and so on^2–4^. Dehumidification using hollow fiber membrane belongs to the category of membrane liquid dehumidification. It combines membrane separation technology with liquid dehumidification technology, which can effectively prevent direct constant between high humidity air and desiccant and eliminate the possibility of mutual pollution between desiccant and air^5,6^. Hollow fiber membrane material is an important part to determine the dehumidification efficiency. Its performance is mainly reflected in selectivity, permeability and its own structure^7–9^. So it is of practical significance to study the characteristics of hollow fiber membrane material to promote the development of this technology.

Porosity refers to the size of microspore volume (or area) contained per unit membrane volume (or area)^10^, which is one of the significant structural parameters of hollow fiber membrane^11^. Porosity leads to mass transfer resistance^12,13^ and mass transfer area^14–17^, which further affects membrane flux. And then some scholars took it a step further. Xiang et al.^18^ found by using experimental methods that porosity increased by 35.3% and pure water flux increased by 286.9%. Peng et al.^19^ studied the performance of porous of ceramic membrane elements, and they found that porosity has a great relationship with pure water flux, and the numerical simulation is in good agreement with experimental results. Liu et al.^20^ showed the humidification efficiency of the membrane liquid dehumidifier increased significantly when the porosity of the fiber membrane changes from 0.1 to 0.5. Wang^21^ used numerical simulation methods to found that the dehumidification efficiency would increase with the increase of porosity. To sum up, previous studies showed that porosity is an important parameter affecting the humidification performance of hollow fiber membrane, but they did not point out the specific law of porosity and humidification amount.

In order to explore the specific law of porosity and humidification performance, a numerical calculation model of countercurrent hollow fiber membrane humidification component was established in this paper. The humidification performance of polypropylene hollow fiber membrane with porosity of 0.45 was analyzed experimentally and the numerical results were verified. Then, by changing the porosity of hollow fiber membrane material in Fluent, the moisture content of the humidified air was analyzed. The relationship between the porosity of hollow fiber membrane material and humidification efficiency was obtained, and a reasonable porosity range of hollow fiber membrane was proposed, which laid a theoretical foundation for the future development of polypropylene hollow fiber membrane material.

## Theoretical basis

**Figure 1:**
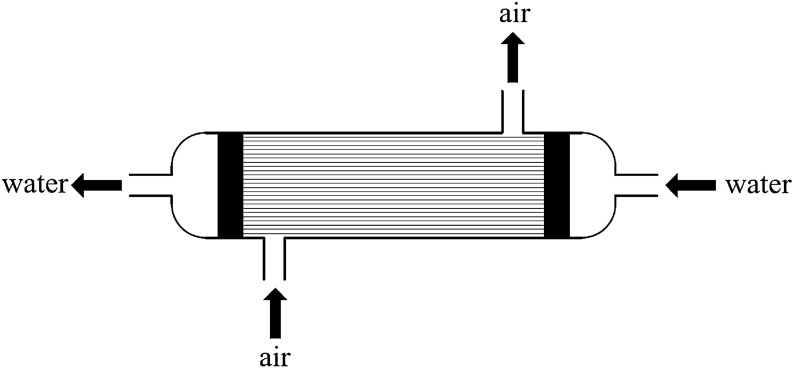
Schematic diagram of countercurrent hollow fiber membrane humidifier component.

**Figure 2:**
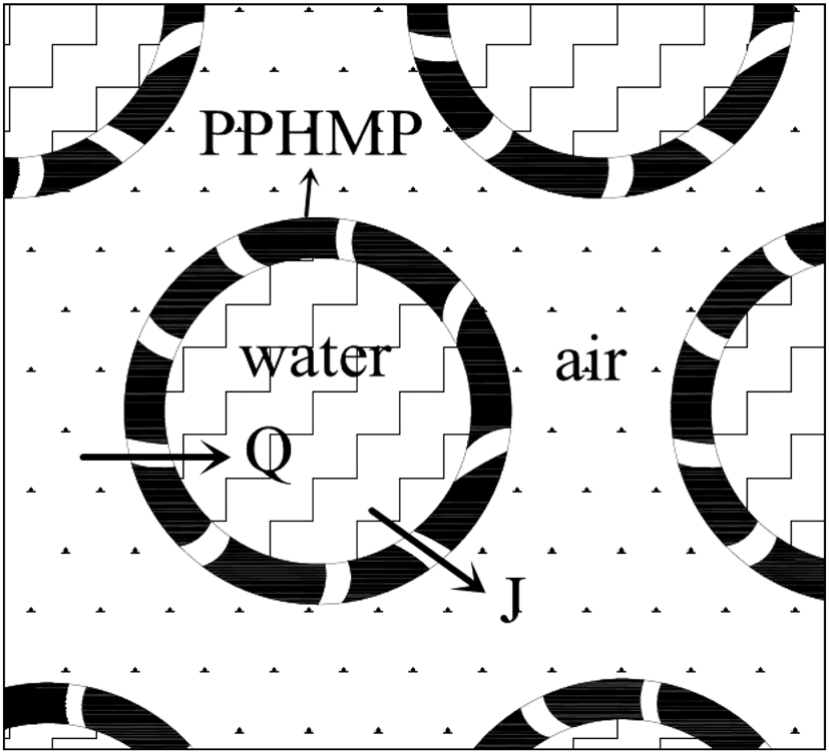
Schematic diagram of countercurrent hollow fiber membrane humidification process.

In this paper, countercurrent hollow fiber membrane module was used for humidification research. When this module works, water vapor molecules reach gas measurement from solution side through the gap between fibers, which is a particularly complex heat and mass transfer process. Figures 1 and 2 are schematic diagrams of humidification of countercurrent hollow fiber membrane assembly.

After years of research by researchers, the heat and mass transfer process is simplified to the exchange of heat and water vapor in air–membrane, membrane–membrane and membrane–solution three regions^22–25^. The heat on both sides of the gas and liquid is mainly transmitted by thermal convection^26–28^. Heat transfer in the film is composed of heat conduction and latent heat of vaporization^29^. The calculation formula of the total heat transfer coefficient is shown in Eq. (1).

1 $$\frac{1}{h}\text{=}\frac{1}{{h}_{1}}+\frac{1}{{h}_{m}}+\frac{1}{{h}_{2}}$$

where *h*_1_ is the heat transfer coefficient of the solution side (W/(m^2^ K)), *h*_2_ is the heat transfer coefficient of the air side (W/(m^2^ K)), *h*_m_ is the heat transfer coefficient of hollow fiber membrane (W/(m^2^ K)).

Heat transfer can be expressed as Eq. (2)

2 $$Q=hA\Delta T$$

where *A* is heat transfer area (m^2^), *∆T* is the logarithmic average temperature difference between air side and solution side (K).

In the process of humidification, mass transfer and heat transfer occur simultaneously in three areas. The humidification amount was mainly discussed in this paper, which refers to the amount of water vapor absorbed by the air in unit time. Its calculation formula^30^ is as follows:

3 $$M=G\mathrm{C}\left({d}_{\mathrm{out}}-{d}_{\mathrm{in}}\right)$$

where *G* is air flow (kg/h), C is the insurance coefficient, which is 1.1 in this paper, *d*_out_ is the moisture content of air outlet (g/kg), *d*_in_ is moisture content of air inlet (g/kg).

The calculation formula of humidification efficiency^30^ is as follows:

4 $$\eta =\frac{{d}_{\mathrm{out}}-{d}_{\mathrm{in}}}{{d}_{\mathrm{e}}-{d}_{\mathrm{out}}}$$

5 $${d}_{\mathrm{e}}=\frac{622{P}_{\mathrm{q}}}{\mathrm{B}-{P}_{\mathrm{q}}}$$

where *d*_e_ is equivalent moisture content (g/kg), *P*_q_ is the partial pressure of water vapor at gas–liquid equilibrium (Pa), B is atmospheric pressure (Pa).

## Numerical simulation

### Assumptions

The heat and mass transfer process of countercurrent hollow fiber membrane humidifier component is complex and easily affected by the external environment. In order to ensure the accuracy of the experiment and simulation, the following hypotheses were made in this paper:

1. All flows are laminar flows, and the liquid is Newtonian fluid;
2. Air is an ideal gaseous mixture of water vapor and dry air;
3. Hollow fiber membrane is homogeneous material and porous medium;
4. Air and solution are evenly distributed in their respective channels and are fully developed fluids;
5. The whole humidifier component is adiabatic with the surrounding environment, and heat transfer only occurs inside;
6. Ignore the heat and mass transfer generated along the flow direction;
7. Heat and mass transfer are steady-state.

### Physical model

**Figure 3:**
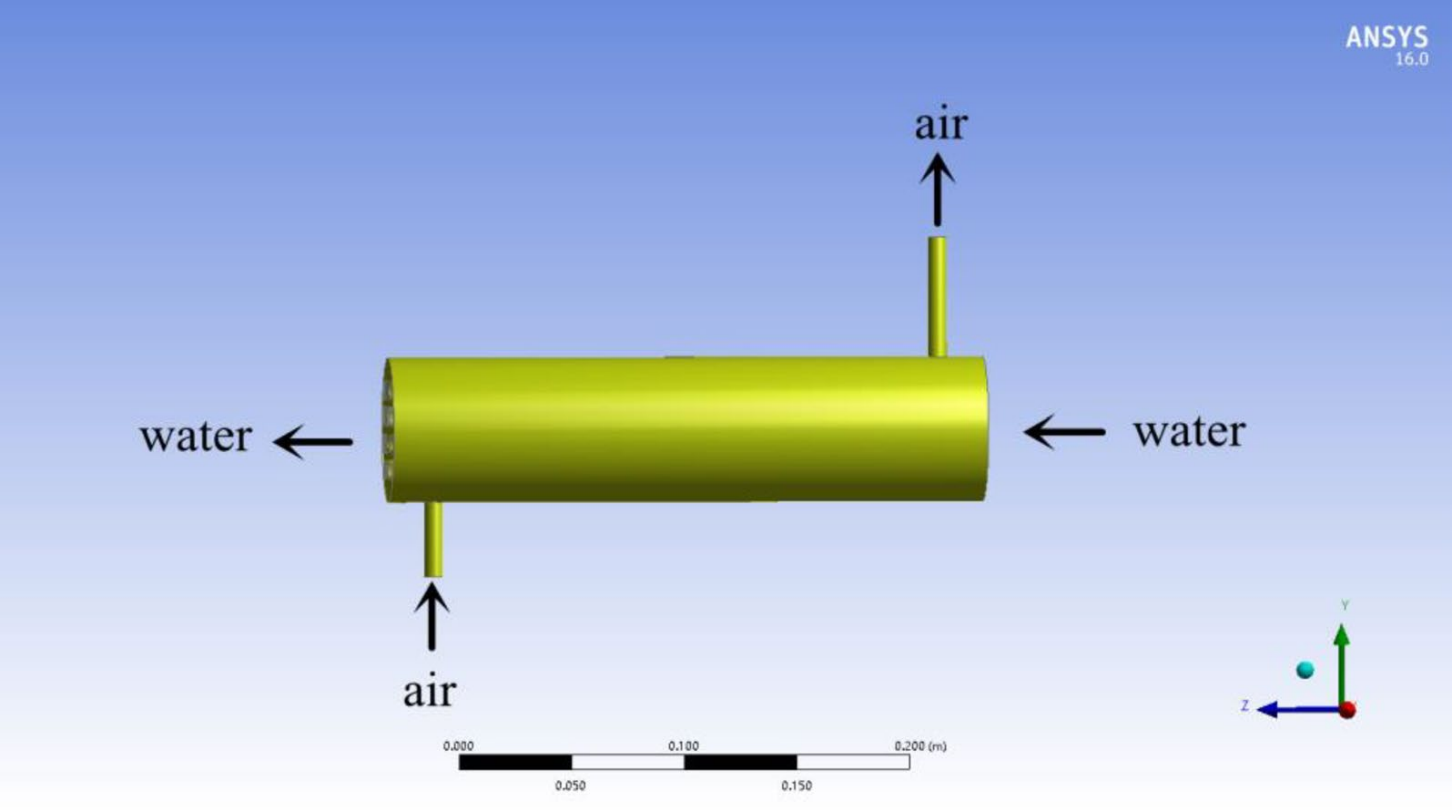
Physical model of counter-flow hollow fiber membrane humidification module (ANSYS 16.0).

Porosity directly affects the flux of the membrane material in the humidification process and the support strength of the membrane material. Membrane flux is proportional to porosity. The support strength is inversely proportional to porosity^31^. Excessive porosity will reduce the support of the fiber membrane and shorten its service life. Generally, the porosity of hollow fiber membrane is between 0.35 and 0.9^32^. In this paper, countercurrent hollow fiber membrane humidifier components with porosity of 0.35, 0.45, 0.65, 0.8, 0.85 and 0.9 were set for simulation. Table 1 lists component parameters. Countercurrent hollow fiber membrane assembly is mainly composed of shell and hollow fiber tube. When it works, the air enters from the lower side of the shell and exits from the upper side, and the solution enters through the right entrance of the fiber tube and exits through the left exit, as shown in Fig. 3.

**Table 1:** Parameters of the hollow fiber membrane humidifier component.

Component parameters	Symbol	Data	Unit
Membrane assembly diameter	D	56	mm
Length of membrane assembly	L	265	mm
Inner diameter of hollow fiber membrane tube	di	0.25	mm
Outer diameter of hollow fiber membrane tube	do	0.4	mm
Pore size	s	0.2	μm
Wall thickness	σ	50	μm
Hollow fiber membrane tube porosity	$\varepsilon$	0.45	
Effective length	Lm	225	mm
Length of shell inlet and outlet pipe	Ls	20	mm
Shell inlet and outlet diameter	dw	13	mm
Component fill rate	χ	0.5	-

### Boundary conditions

In order to explore the relationship between porosity and humidification performance, the humidification process of hollow fiber membrane with six porosity values was simulated under five air flow conditions (Table 2). The solution was completed in Fluent16.0, and related parameter Settings are shown in Table 3.

**Table 2:** Air condition.

Air condition	Condition 1	Condition 2	Condition 3	Condition 4	Condition 5
Air flow rate (kg/h)	2.96	5.92	8.88	11.84	14.80
Liquid flow rate (kg/h)	7.00	7.00	7.00	7.00	7.00
Temperature at the air inlet (K)	298	298	298	298	298
Temperature at the inlet of the liquid (K)	290	290	290	290	290
Moisture content at the air inlet (g/kg)	4.9	4.9	4.9	4.9	4.9

**Table 3:** Parameter settings of the numerical simulation.

Parameter	Setting
Flowing model	Laminar
Fluid	Air, water, water vapor
Solid	Porous membrane material, porosity 0.35/0.45/0.65/0.8/0.85/0.9
Inlet of air	Velocity-inlet (calculated according to the air flow in Table 2) Relative humidity 25% Inlet temperature 298 K Moisture content 4.9 g/kg
Outlet of air	Pressure-out
Inlet of liquid	Velocity-inlet (calculated according to the solution flow in Table 2)
Out of liquid	Pressure-out

## Results and discussion

### Experimental verification

**Figure 4:**
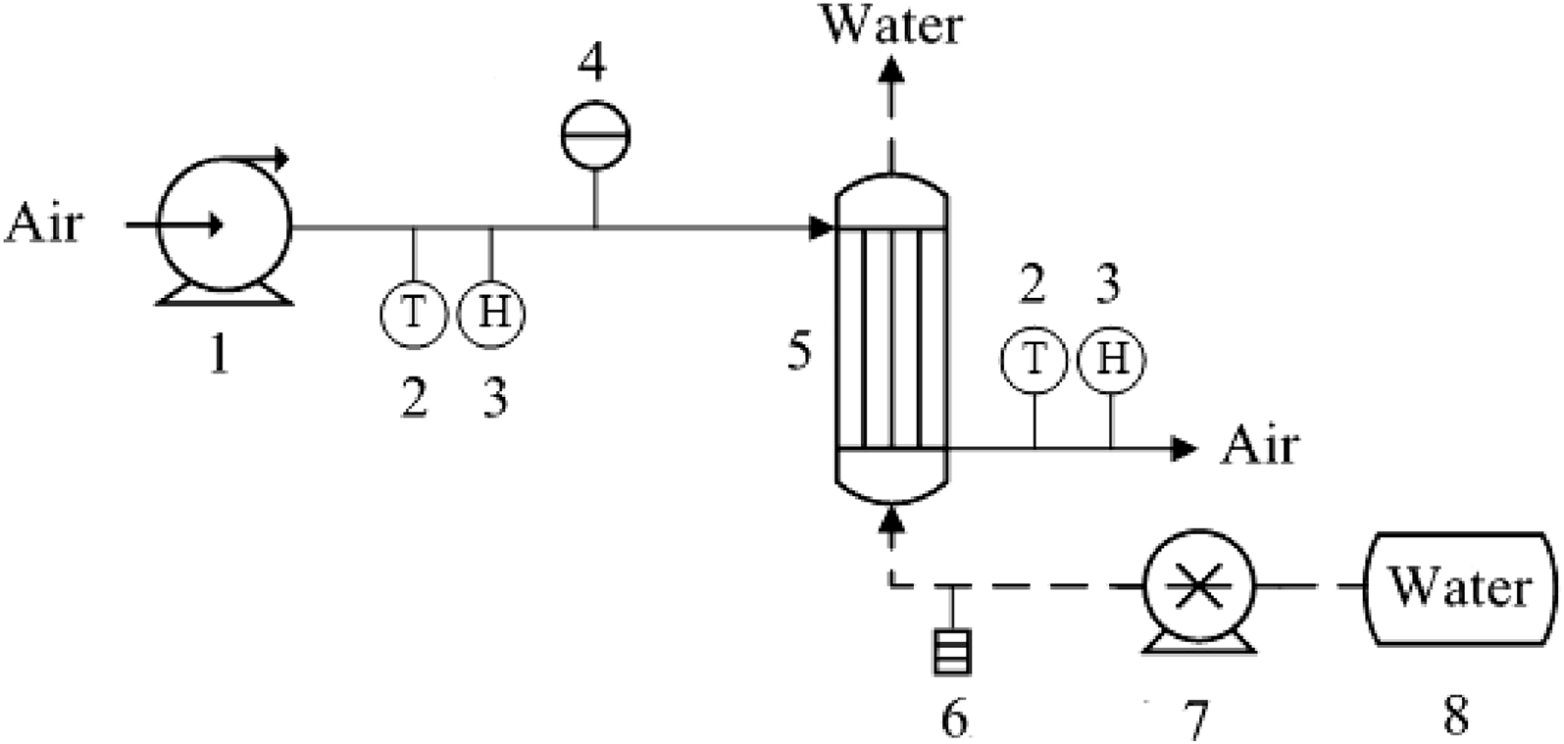
Experiment service schematic diagram (1. Air pump, 2. Temperature tester, 3. Hygrometer, 4. Hot-wire anemometer, 5. Hollow fiber membrane humidifier component, 6. Rotameter, 7. Solution pump, 8. Constant temperature water tank).

In order to ensure the correctness of numerical simulation, an experimental system of polypropylene hollow fiber membrane humidification component was built according to the relevant parameters shown in Table 1, and the schematic diagram is shown in Fig. 4. The experimental system consisted of an air loop and a solution loop. The air loop comprised air compressor, air duct, hot-wires anemometer, temperature and humidity tester, and shell of hollow fiber membrane humidifier component. The temperature and humidity of the air in the experiment were regulated by the air conditioning equipment in the room. The air compressor adjusted the air flow rate by controlling the pneumatic valve, and sended the air into the hollow fiber membrane humidification component to provide power for the air circulation system. The solution loop consisted of constant temperature water tank, solution pump, rotameter, solution pipeline, and valve. The solution pump feeded the solution in the constant temperature tank into the hollow fiber membrane humidifier, and the solution flow rate was controlled by the solution pump. The solution that is sent into the hollow fiber membrane tube completed the heat and mass exchange with the air and then returns to the constant temperature flume again after treatment. The circulation of aqueous solution was realized.

Due to the limitation of experimental conditions, only hollow fiber membrane with porosity of 0.45 was verified. The numerical simulation results and experimental results are shown in the Table 4. The results have a small margin of error, within 5%.

**Table 4:** Data statistics.

Air condition	Temperature at the air outlet (K)			Humidity at the air outlet (g/kg)		
	Numerical simulation results	Experimental results	Result error (%)	Numerical simulation results	Experimental results	Result error (%)
Condition 1	294.8	295.1	0.11	11.4	11.1	2.70
Condition 2	295.5	295.8	0.10	10.9	10.8	0.93
Condition 3	296.2	296.1	0.03	10.4	10.6	1.89
Condition 4	296.5	296.3	0.06	10.3	10.5	1.90
Condition 5	297.0	296.5	0.17	10.2	10.1	0.99

**Figure 5:**
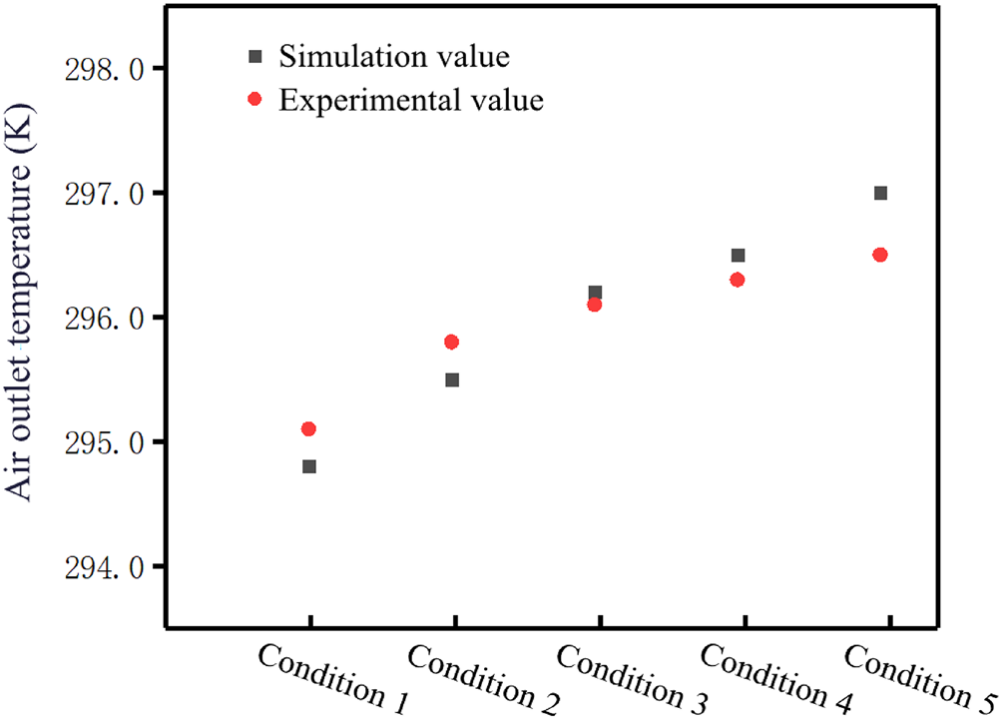
Comparison of experimental and simulated air outlet temperature under different working conditions (Origin 2018).

**Figure 6:**
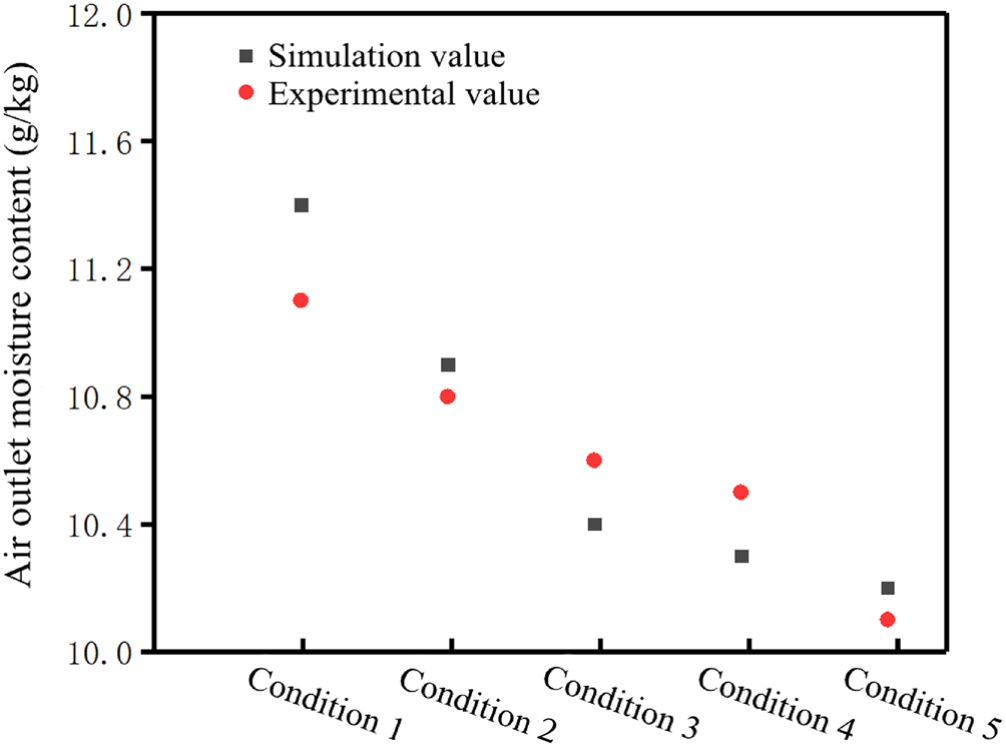
Comparison between experimental value and simulation value of air outlet moisture content under different working conditions (Origin 2018).

The simulation results were consistent with the experimental results as the air flow increases. As the air flow gradually increased, the temperature at the air outlet side increased (Fig. 5) and the moisture content decreased (Fig. 6). To sum up, the numerical simulation results obtained in this paper are reliable.

### Results and analysis

**Figure 7:**
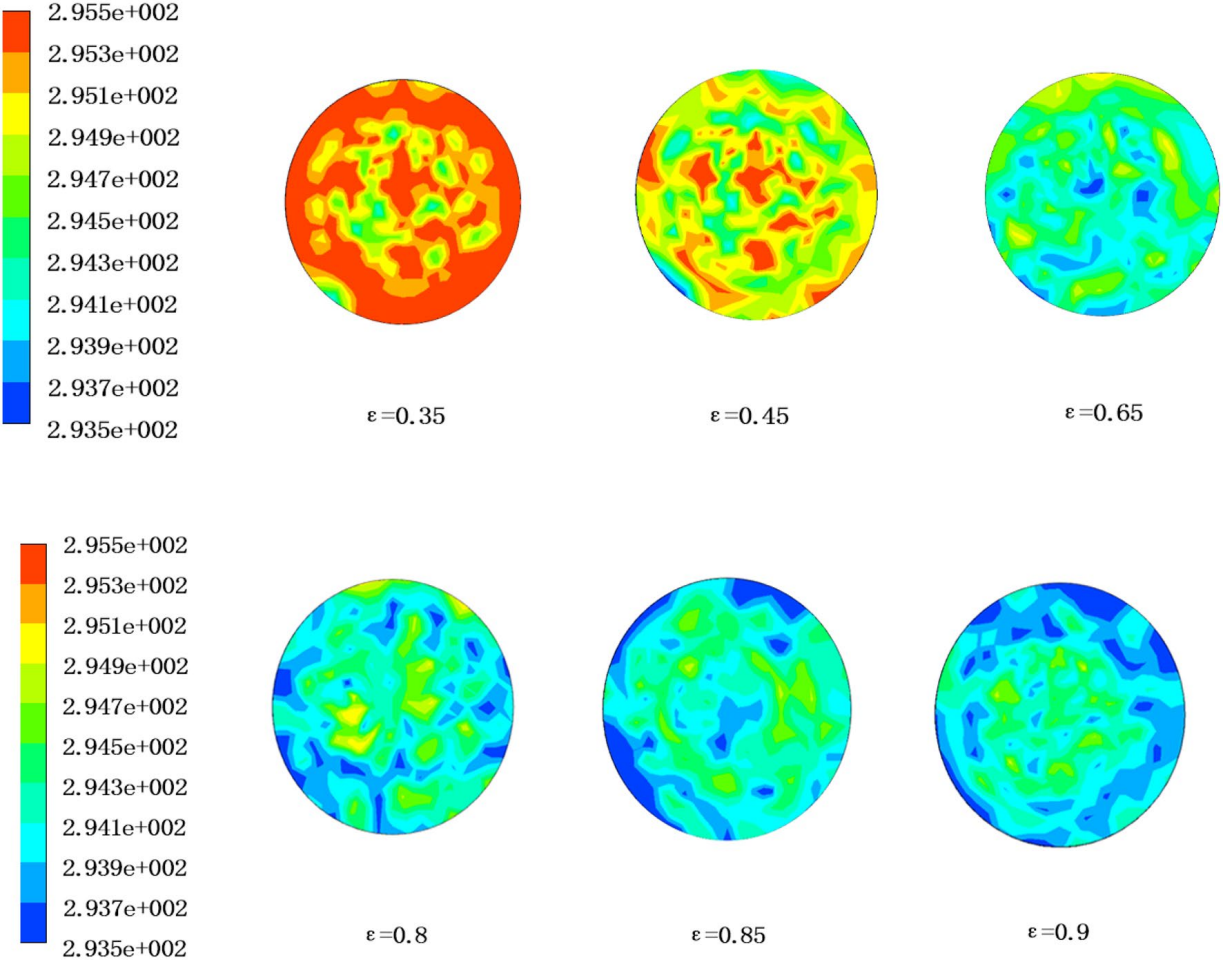
Air outlet temperature distribution under different membrane porosity (ANSYS 16.0).

In order to study the effect of porosity on humidification performance of countercurrent hollow fiber membrane assembly, the numerical simulation results were analyzed as follows. Figure 7 shows the temperature distribution cloud diagram at the air outlet side of the 6 porosity of the hollow fiber membranes under working condition 1. It can be seen from the figure that with the increase of porosity, the temperature at the air outlet side gradually decreased. This is because the increase of porosity enhances the heat transfer efficiency on both sides of the film, so that more heat is transferred from the air to the solution, resulting in lower and lower temperature at the air outlet side.

**Figure 8:**
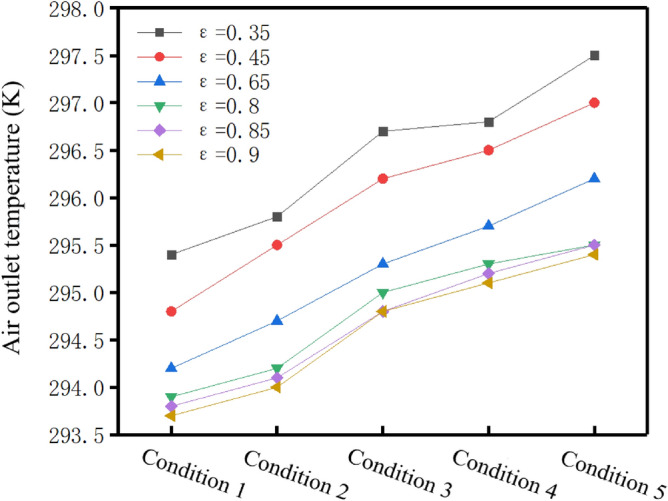
Change of air outlet temperature with air flow rate under different porosity (Origin 2018).

Figure 8 shows the variation of air outlet temperature of the 6 porosity of hollow fiber membranes with air flow under 5 working conditions. It can be seen that under the condition of constant porosity, the air outlet side temperature increased with the increase of air flow. This is because the increase of air flow reduces the air contact time with the fiber membrane and thus shorten the heat transfer time. In addition, under constant air flow condition, the air outlet temperature decreased with the increase of porosity. When the porosity changes from 0.35 to 0.8, there was an obvious temperature difference of about 2 K at the air outlet side. However, when the porosity changed from 0.8 to 0.9, the temperature difference at the air outlet side was only about 0.2 K. There was no obvious temperature change. This phenomenon indicates that when the porosity was between 0.35 and 0.8, the heat transfer effect of the hollow fiber membrane humidifier component was significantly enhanced. However, when the porosity was between 0.8 and 0.9, the increase of porosity had no obvious effect on enhancing the heat transfer effect of humidifier components.

**Figure 9:**
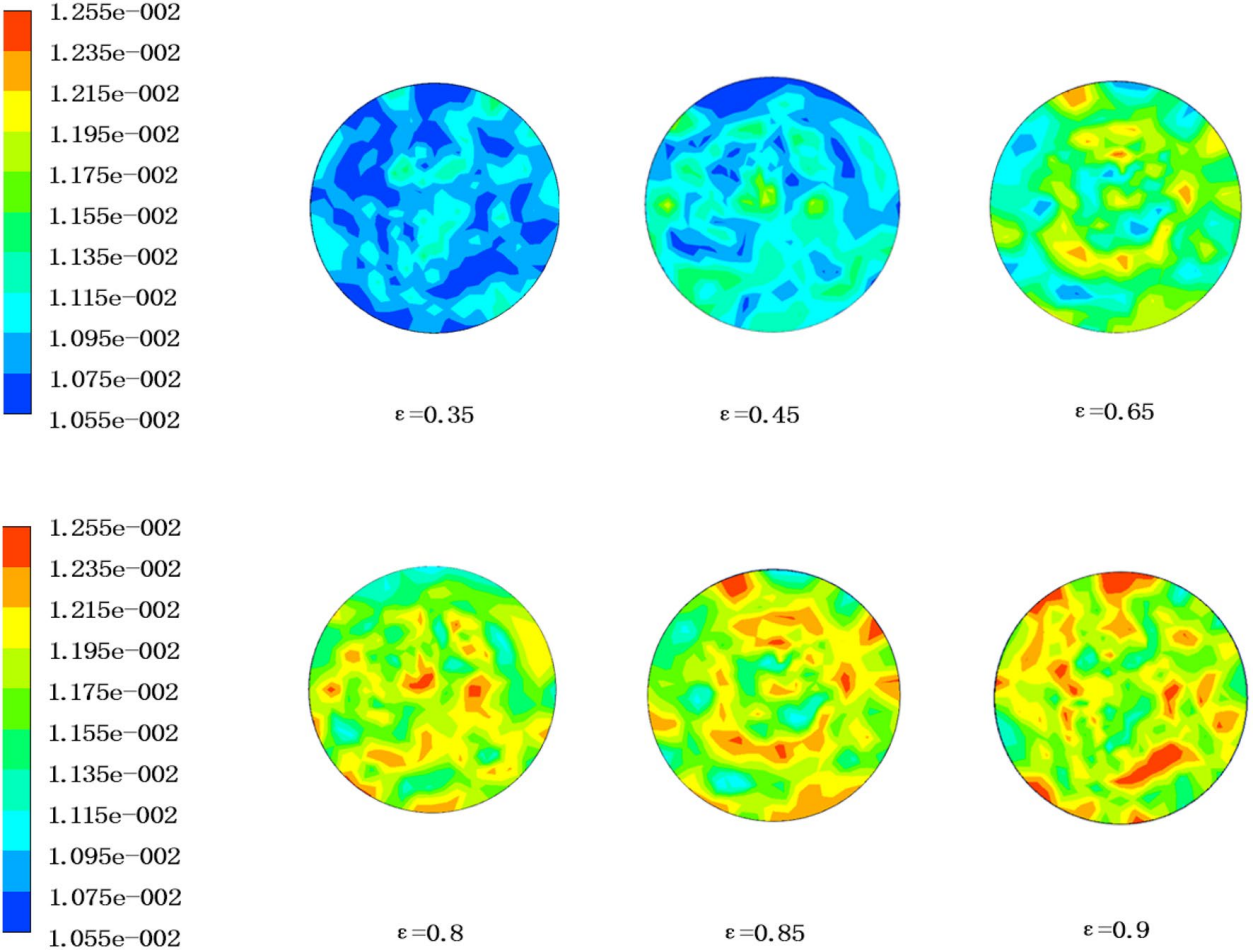
Distribution of water vapor mass fraction at air outlet with different membrane porosity (ANSYS 16.0).

Figure 9 shows the water vapor mass fraction distribution cloud diagram at the air outlet side of the 6 porosity of the hollow fiber membranes under working condition 1. It can be seen that as the porosity gradually increased, there were more and more water vapor molecules on the side of the air outlet, which also indicated that the moisture content of the air was getting higher and higher. This is because the increase of porosity enhanced the mass transfer process and increases the transmembrane flux, thus increasing the moisture content of the air outlet side.

**Figure 10:**
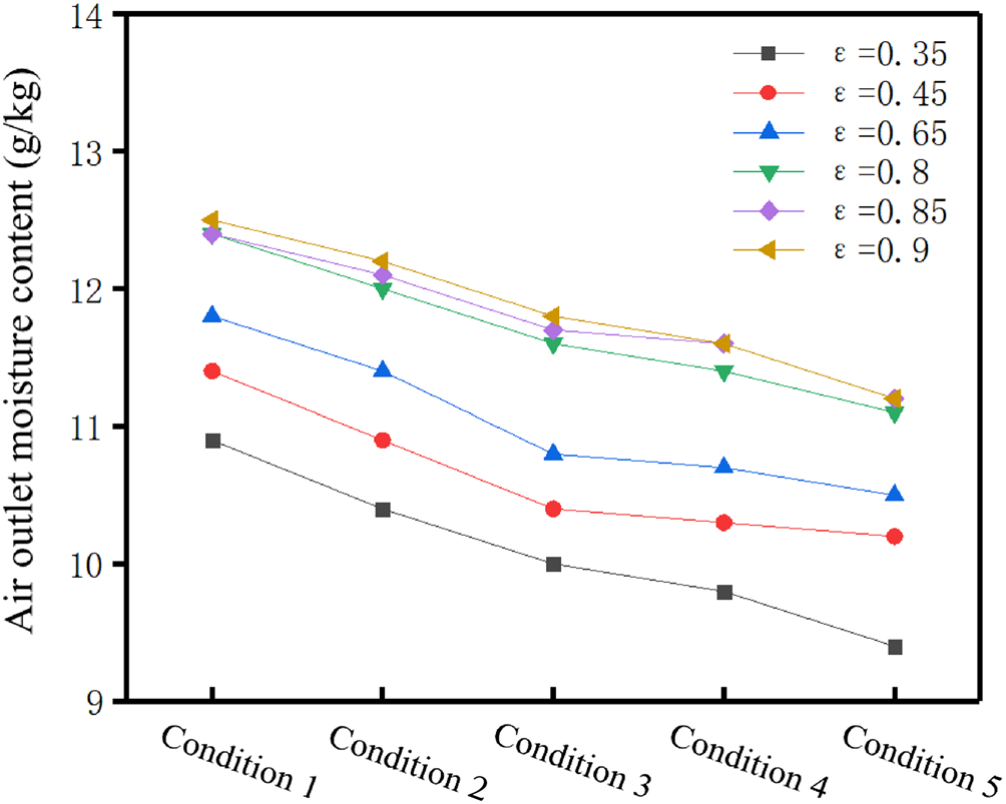
Change of air outlet moisture content with air flow rate under different porosity (Origin 2018).

Figure 10 shows the variation of air outlet moisture content of the 6 porosity of hollow fiber membranes with air flow under 5 working conditions. It can be seen that under the condition of constant porosity, the moisture content at the air outlet side was decrease with the increase of air flow. This is because the increase of air flow reduced the mass transfer driving force between air and solution, leading to the decrease of air outlet moisture content. In addition, under the condition of constant air flow rate, the moisture content of air outlet increases with the increase of porosity. When the porosity varied from 0.35 to 0.8, the moisture content at the air outlet side increased obviously about 1.5 g/kg. However, when the porosity changes from 0.8 to 0.9, the moisture content at the air outlet side didn’t increase significantly, and even does not increase when the porosity changes from 0.8 to 0.85 at the first and fifth working conditions. This indicates that when the porosity was 0.35–0.8, the mass transfer effect of hollow fiber membrane is significantly improved, while when the porosity is 0.8–0.9, the mass transfer effect is not significantly enhanced.

**Figure 11:**
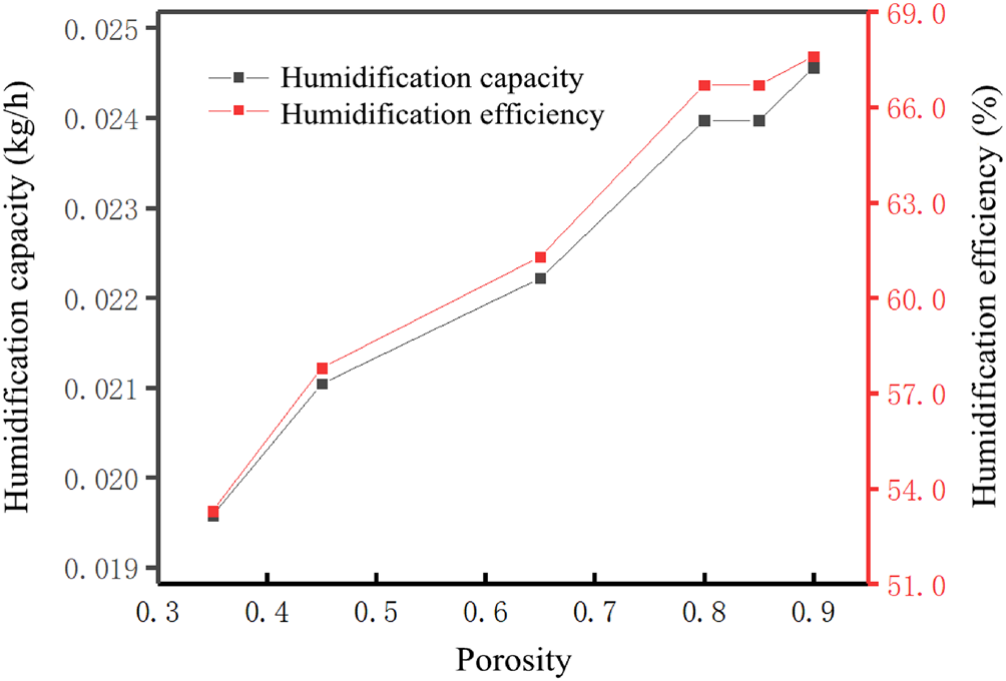
Humidification capacity and humidification efficiency under different porosity under working condition one (Origin 2018).

Figure 11 shows the variation of humidification amount and efficiency with porosity under condition 1. It can be seen that when the porosity increases from 0.35 to 0.8, the humidification amount increased from 0.0193 to 0.0242 kg/h, and the humidification efficiency increased from 53.3% to 66.7%. The humidification amount and efficiency increase significantly with the increase of porosity. However, when the porosity increased from 0.8 to 0.85, the humidification amount and efficiency hardly change, indicating that the increase of porosity has no obvious effect on improving the humidification amount and efficiency. When the porosity increased from 0.8 to 0.9, the humidification capacity increased from 0.0242 to 0.0248 kg/h, and the humidification efficiency increased from 66.7 to 67.6%. Although the increase of porosity also increases the humidification capacity and efficiency, compared with the increase of porosity from 0.35 to 0.8, the increase of porosity has no significant effect on the improvement of humidification capacity and efficiency. This result is obtained through the analysis of the right amount and humidification efficiency under all air flow conditions, so it will not be described too much here.

## Conclusion

Through the study of the performance of hollow fiber membrane humidification system made of porous materials, the following conclusions are drawn:

1. Under the condition of constant porosity, the increase of air flow reduced the contact time between air and solution per unit volume, resulting in the decrease of heat transfer effect, and the air outlet temperature increases with the increase of air flow. Similarly, the increase of air flow leaded to the decrease of moisture content at the air outlet side, which reduced the humidification efficiency.
2. Under constant flow condition, the air outlet temperature decreases with the increase of porosity, indicating that porosity strengthens the heat transfer effect of hollow fiber membrane humidifier. The moisture content at the air outlet side increases with the increase of porosity, indicating that the greater the porosity, the greater the membrane flux and the greater the humidification.
3. Heat and mass transfer performance of hollow fiber membrane humidifier component shows an upward trend with porosity. When the porosity increases from 0.35 to 0.8, the increment of heat and mass transfer is obvious, but when the porosity is greater than 0.8, the increment of heat and mass transfer performance is not obvious. The increase of porosity will reduce the mechanical strength of hollow fiber membrane humidifier components to a certain extent and affect the service life. Taking the above factors into consideration. When the porosity is between 0.65 and 0.8, the hollow fiber membrane humidifier can not only ensure high heat and transfer quality, but also ensure its mechanical strength to a certain extent.
