# Author Correction: Study on the effect of porosity of hollow fiber membrane on humidification performance

**DOI:** 10.1038/s41598-022-09750-4

**Published:** 2022-04-04

**Authors:** Runping Niu, Xiaoting Jia, Lizhi Geng

**Affiliations:** School of Environment and Energy Engineering, Beijing University of Civil Engineering and Architecture, Beijing, 100044 China

Correction to: *Scientific Reports* 10.1038/s41598-022-07869-y, published online 09 March 2022

In the original version of this Article Runping Niu, Xiaoting Jia and Lizhi Geng were incorrectly affiliated with ‘Engineering and Architecture, Beijing University of Civil, Beijing 100044, China’. The correct affiliation is listed below.

School of Environment and Energy Engineering, Beijing University of Civil Engineering and Architecture, Beijing, 100044, China.

The original Article has been corrected.

